# Efficacy of Hydroxytyrosol-Rich Food Supplements on Reducing Lipid Oxidation in Humans

**DOI:** 10.3390/ijms24065521

**Published:** 2023-03-14

**Authors:** Cecilia Bender, Ilaria Candi, Eva Rogel

**Affiliations:** 1 Institut Kurz GmbH, 50829 Köln, Germany; 2 Istituto Kurz Italia S.R.L., 43126 Parma, Italy

**Keywords:** olive phenolics, hydroxytyrosol, oxidized LDL, oxLDL, isoprostanes, F2-isoprostanes

## Abstract

In the present study we report the efficacy of two food supplements derived from olives in reducing lipid oxidation. To this end, 12 healthy volunteers received a single dose (25 mL) of olive phenolics, mainly hydroxytyrosol (HT), provided as a liquid dietary supplement (30.6 or 61.5 mg HT), followed by an investigation of two reliable markers of oxidative stress. Blood and urine samples were collected at baseline and at 0.5, 1, 1.5, 2, 4, and 12 h post-intake. Plasma-oxidized low-density lipoprotein (oxLDL) cholesterol levels were measured with ELISA using a monoclonal antibody, while F2-isoprostanes (F2-IsoPs) were quantified in urine with UHPLC-DAD-MS/MS. Despite the great variability observed between individuals, a tendency to reduce lipoxidation reactions was observed in the blood in response to a single intake of the food supplements. In addition, the subgroup of individuals with the highest baseline oxLDL level showed a significant (*p* < 0.05) decrease in F2-IsoPs at 0.5 and 12 h post-intervention. These promising results suggest that HT supplementation could be a useful aid in preventing lipoxidation. Additionally, people with a redox imbalance could benefit even more from supplementing with bioavailable HT.

## 1. Introduction

An altered balance of free radicals, resulting in high levels of oxidative stress, is associated with many chronic human diseases such as cardiovascular diseases including atherosclerosis, which are a leading cause of global mortality [1,2,3]. The first biochemical event in atherosclerosis is the oxidation of LDL in the vascular wall, producing oxLDL [4], which is involved in the formation and progression of atherosclerotic plaque [5,6]. The oxidative process of the LDL consists of a chemical modification of the protein’s moiety through the myeloperoxidase-derived enzyme [7] or the oxidation of LDL’s polyunsaturated fatty acids (PUFA) [8]. Nutrition plays an important role in the prevention of atherosclerosis [9]; in particular, dietary antioxidants can protect LDL from oxidation [10,11].

The olive tree contains phenolic compounds such as HT, tyrosol (Tyr), and oleuropein (Ole) [12,13], which have key roles in plant physiology such as enhancing the resistance to insects and microorganisms [14]. The oil obtained from the pressing of olive fruits contains phenolic compounds that contribute to the protection of LDL from oxidation. Different qualities of olive oils are available on the market, from which only extra virgin olive oil (EVOO), the less-processed olive oil that is obtained from milling and cold pressing, may contain enough phenolics [15,16] to protect LDL from oxidation.

In particular, HT, one of the main antioxidants in olives, is also believed to act in table olives and oil, and has received health claim approval in the EU [17]. Indeed, the European Food Safety Authority (EFSA) has confirmed that 20 g of olive oil containing at least 5 mg of HT and its derivatives (Tyr and Ole complexes) contribute to the protection of LDL from oxidation.

Despite the belief that a diet rich in olive oil should guarantee an adequate intake of olive phenolics, which due to their antioxidant capacity would have a positive effect on the prevention of cardiovascular risks by preventing LDL oxidation, other evidence suggests that the daily intake of HT, Tyr, and phenolic compounds would be insufficient [18,19] to obtain the desired physiological effect. Because these compounds are present in the olive fruit, they are also found in olive derivatives such as olive oil and vegetation waters. And because many of these substances are hydrophilic, they are found to a greater extent in the aqueous fraction that results from pressing the olive fruit during oil production [20]. It seems that to ensure the minimum useful daily dose for the protection of LDL, it would be necessary to enrich the olive oil with HT; alternatively, the consumption of food supplements containing bioavailable HT would be a valid resource.

In this context, we recently reported the cellular antioxidant properties of two dietary supplements produced using the vegetation water generated during olive oil production [20], one with the addition of 6% lemon juice and the other with 70% grape juice. The results derived from this preliminary study denoted an important antioxidant potential of both supplements in cellular models. More recently, in a randomized controlled clinical study, we showed that dietary HT provided with both food supplements is absorbed from the intestinal tract in variable amounts and is rapidly metabolized into phase I and phase II metabolites [21]. The main HT metabolites, which were not found in the fasting state, were rapidly cleared from the plasma in the postprandial phase (t_max_ 30 min, complete clearance 2–4 h) and excreted in the urine mainly as 3,4-dihydroxyphenylacetic acid, homovanillic acid, and hydroxytyrosol sulfate [21].

However, the effect of supplementation with olive phenolics on lipoxidation markers has been little considered in foods other than olive oil. Therefore, the aim of this study was to investigate the effect of commercially available olive-derived dietary supplements on lipoxidation markers in humans. We hypothesized that these food supplements rich in natural bioavailable HT may strengthen the antioxidant capacity in the postprandial phase; thus, we here report the secondary outcome of this clinical study by measuring the kinetic of two reliable markers of lipoxidation, oxLDL and F2-IsoPs.

F2-IsoPs are prostaglandin-like lipid peroxidation products of arachidonic acid, the main PUFA present in human cells. Numerous studies have shown these molecules to be accurate markers for systemic oxidative damage in plasma or urine [22,23,24] and their decrease to indicate a lower in vivo peroxidation of lipids.

Our data indicate that a single intake of HT through both dietary supplements tends to reduce the mean concentration of oxLDL in the blood. In addition, the F2-IsoPs measurement from subjects whose oxLDL levels were elevated at baseline shows that the intake of either food supplement significantly reduces the F2-IsoPs content in urine.

Despite the small sample size, the results obtained here are promising, especially for the balance of redox status in the body. Quite interesting is the marked decrease of both biomarkers of oxidative stress as a result of taking these HT-rich food supplements, with the caveat that the effect depends on the individual’s baseline condition. People who have high starting levels of these markers will experience a higher benefit.

## 2. Results

### 2.1. Characterization of the Food Supplements

The efficacy of watery food supplements derived from olive vegetation water was investigated in relation to the modulation/reduction of lipid oxidation in humans. For this, a randomized, blind, crossover study was carried out with 12 healthy volunteers who received acute supplementation of *Oliphenolia bitter* (hereafter referred to as IP-1), which is composed of 94% concentrated vegetation water and 6% concentrated lemon juice, or *Oliphenolia* (hereinafter IP-2), which is composed of 30% further concentrated vegetation water and 70% concentrated grape juice. The administered dose (25 mL – 1 flask) of the food supplements contained HT as the main bioactive (Table 1) together with (poly)phenols naturally derived from olives and lemons or grapes for IP-1 and IP-2, respectively.

One volunteer dropped out of the study after the completion of the first intervention period (IP-2) and was subsequently replaced. Considering the data from this subject, the sample size for the IP-2 group is 13. The overall intervention sample size is 25 (as the sum of IP-1 and IP-2).

### 2.2. Plasma oxLDL

**Figure 1:**
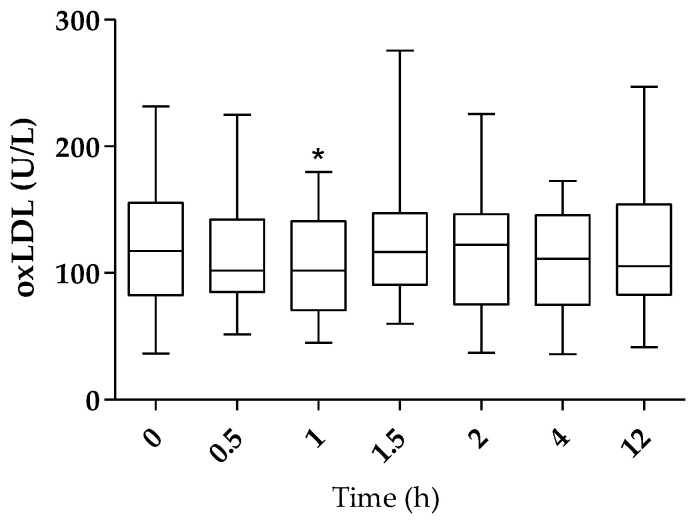
oxLDL values before and after HT administration through food supplements (n = 25). The graph shows the median oxLDL concentration (U/L) in plasma, maximum and minimum values, 25% and 75% percentiles. *: significantly different from baseline with *p* < 0.05.

Figure 1 shows box plots representing the oxLDL content pre-intervention and up to 12 h after intake (as the sum of IP-1 and IP-2, n = 25). The baseline plasma oxLDL levels resulting were highly variable between subjects (125.5 ± 10.49 U/L), as well as highly intra-variable (data not shown). As shown in Figure 1, a downward trend in oxLDL levels was observed after ingestion for up to 12 h, although this trend reached statistical significance after 1 h only (108.0 ± 8.66 U/L, *p* < 0.05).

**Figure 2:**
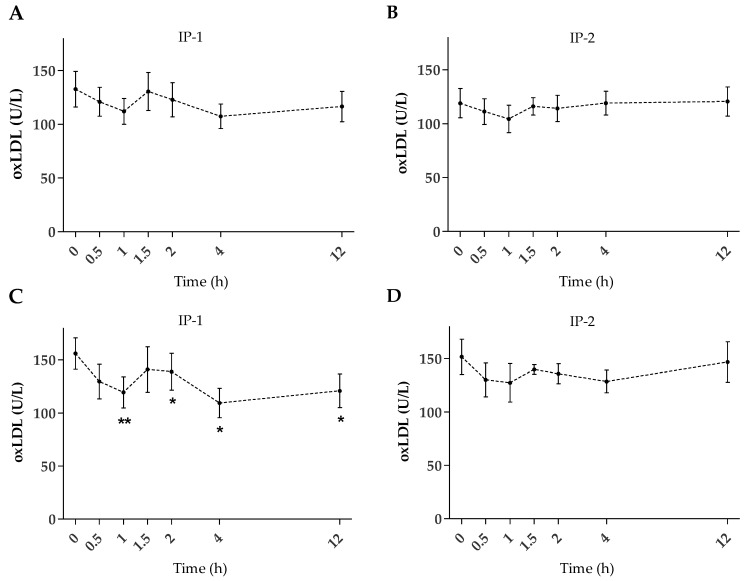
Mean plasma oxLDL concentration over time before (0 h) and after dietary interventions. (**A**) IP-1 (n = 12), (**B**) IP-2 (n = 13), and using the highest oxLDL concentration at baseline (**C**) IP-1 (n = 9), and (**D**) IP-2 (n = 7). Bars: SEM. Significantly different from baseline with *: *p* < 0.05, and **: *p* < 0.01.

The analysis of the average oxLDL of the independent interventions (IP-1 or IP-2) shows a trend toward the reduction of oxLDL post-intervention (Figure 2A,B); however, the small sample size and the wide inter-individual variation observed do not allow to show statistical significance.

Interestingly, when the statistical analysis is stratified to include only the subjects with a high level of oxLDL pre-intervention (cut off 101 U/L) that is, basal mean values higher than reported for healthy subjects [25], the oxLDL level pre-intervention (155.89 ± 14.75 U/L, n = 9) was significantly reduced at 1 h (119.46 ± 14.62 U/L, *p* < 0.01) after intake of IP-1 only (Figure 2C), remaining significantly reduced (*p* < 0.05) at 2, 4, and 12 h post-intervention.

### 2.3. Urinary F2-IsoPs

The degree of oxidative stress was further evaluated by quantifying the F2-IsoPs in the urine in those subjects showing high oxLDL levels at baseline (155.89 ± 14.74 U/L and 151.55 ± 16.54 U/L for IP-1 and IP-2, respectively). Three different F2-IsoPs isomers, namely 8-isoPGF2α, ent-PGF2α, and 2,3-dinor-8-isoPGF2α, were quantified at baseline and after the intake of the food supplements. In response to a single 25 mL intake of either of the food supplements a significant decrease (*p* < 0.05) in F2-IsoPs (as the sum of the three isomers) was shown at 0.5 and 12 h (Table 2). Quantitatively, the excreted amount of F2-IsoPs was higher at baseline compared with after the intake of the food supplements, being more pronounced for IP-2 compared with IP-1.

**Figure 3:**
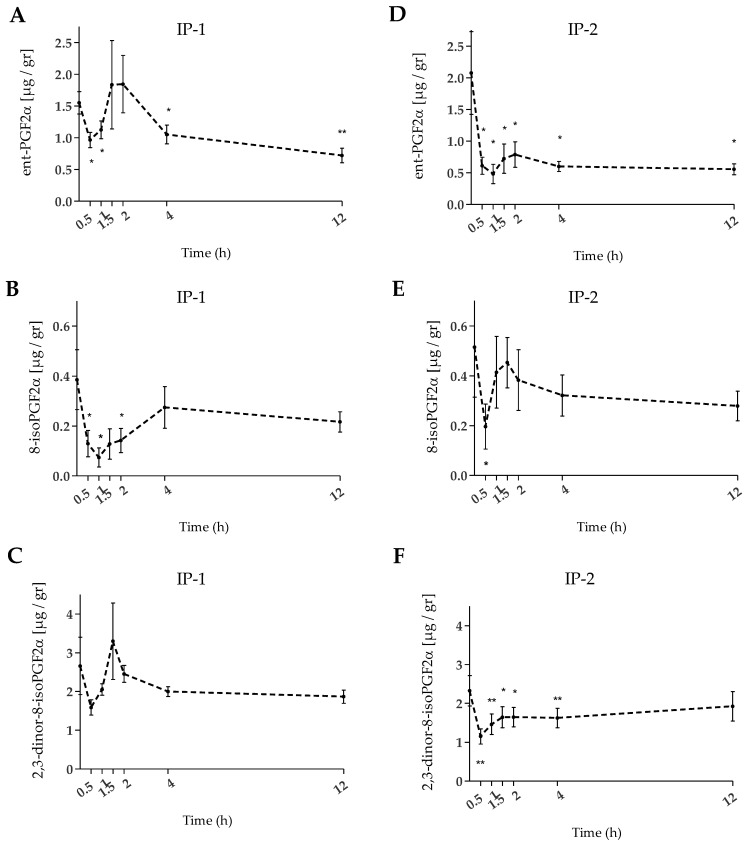
Mean urinary IsoPs content over time, before (0 h) and after ingestion of IP-1 (**A**–**C**) and IP-2 (**D**–**F**). Bars: SEM. Significantly different versus baseline with *: *p* < 0.05, and **: *p* < 0.01.

The isomers 2,3-dinor-8-isoPGF2α and ent-PGF2α were the main ones in terms of absolute concentration in urine; for both, the downward trend was strongest with IP-2 (Figure 3). In contrast, IP-1 better counteracted 8-isoPGF2α levels, although its abundance was ~4 to ~6-fold lower than the isomers ent-PGF2α and 2,3-dinor-8-isoPGF2α, respectively. Notably, changes in the ent-PGF2α isomer exhibited a two-phase kinetic pattern, with a significant decrease early in the time course, followed by an increase between 1.5 and 2 h, then a significant decrease again at 4 and 12 h after intake of the food supplements (Figure 3A,D). A similar pattern was observed for 8-isoPGF2α (Figure 3B, E), where the pre-intervention level decreased significantly after 0.5 h post-intake (*p* < 0.05); for IP-1 only, the reduction was significantly sustained over time up to 2 h. On the other hand, the abundance of 2,3-dinor-8-isoPGF2α was significantly lower (*p* < 0.01) immediately after the intake of IP-2 only (Figure 3F), remaining significantly reduced until 4 h after the intake.

## 3. Discussion

The objective of this crossover study was to verify whether the two watery food supplements, particularly rich in HT from olives, are effective in reducing lipid oxidation in humans. To this aim, plasma oxLDL and urinary F2-IsoPs were chosen as reliable biomarkers.

While previous studies with foods containing olive phenolics supported their role in lowering oxLDL in vivo [26,27,28,29,30,31,32,33,34], these studies were performed mainly with HT delivered in oil and after short- or medium-term interventions; however, to the best of our knowledge, there are no previous reports showing that olive phenolics provided in a watery matrix can also reduce oxLDL in vivo after an acute intake. Our results show a significant reduction in plasma oxLDL as soon as 1 h after the intake of a single dose of the food supplements. We also observed a downward trend in the pre-intervention levels of oxLDL in both supplement groups, studied separately (although this trend did not reach statistical significance). Levels of oxLDL showed a large inter- and intra-variability at baseline, suggesting that baseline values may play a role in the effect of HT on this marker. 

Considering only subjects with high levels of oxLDL at baseline, a significant reduction in oxLDL was observed at 1, 2, 4, and 12 h after the administration of IP-1 (30.6 mg HT + 0.04 mg Ole). Under this condition the performance of IP-1 was superior to that of IP-2.

Unlike what was reported in the literature [27,28,29,30,31] for olive oil intake, our results do not show a simple dose dependence for an oxLDL protective effect. In fact, IP-2, the food supplement containing the highest dose of olive phenolics (61.5 mg HT + 0.07 mg Ole), did not cause a higher oxLDL reduction than IP-1, which contains almost half the HT-dose together with the lemon juice concentrate. This result is in line with our pharmacokinetic study [21], which showed that the excreted percentage of total ingested HT (calculated as the sum of all quantifiable metabolites in 12-h urine) is higher for IP-1, followed by IP-2, and then HT-fortified EVOO to a lesser extent. This finding reinforces the importance of other dietary factors influencing both bioavailability and bioefficacy, such as positive or negative matrix effectors, water or fat content, differences in phenolics either by type or content, as well as synergistic or antagonistic interactions with other food components.

Our results show that plasma oxLDL likely can be lowered in subjects whose level was high at baseline, suggesting that the HT is particularly effective in individuals who are experiencing an oxidative imbalance. This observation is supported by several human trials conducted in subjects with high oxidative stress conditions, in which a significant reduction of lipoxidation was documented using different biomarkers, such as plasma oxLDL or urinary IsoPs [34,35,36,37,38]. In particular, Sarapis [34] recently reported a significant decrease of oxLDL after consumption of a high dose of olive oil polyphenols, a reduction that became more pronounced in subjects with a high cardiometabolic risk.

In 2011, the EFSA published a scientific opinion in which it stated that the daily consumption of at least 5 mg of HT and derivatives (Tyr and Ole) contained in 20 g of olive oil protects the LDL against oxidation [17]. Nevertheless, other researchers pointed out that the daily intake of HT, Tyr, and phenolic compounds through a typical Mediterranean diet would supply only around 2 mg [18]. Moreover, the intake of Tyr and HT from virgin olive oil would be between 88.5 and 237.4 µg daily [19]. These studies seem to indicate that the amount of HT and its derivatives ingested daily with the Mediterranean diet or with olive oil alone would be insufficient to reach the EFSA-stated minimum intake level of 5 mg. Therefore, it seems to be advisable to increase the intake of HT and its derivatives in order to obtain the effect protective of LDL. Such an increase can be better achieved by the consumption of bioavailable HT-containing supplements.

In addition to plasma LDL oxidation, another important marker to consider for the prevention of oxidative processes in vivo is the F2-IsoPs. Few studies conducted with healthy volunteers have evaluated the IsoPs content after ingesting foods derived from olives. The results remain somewhat contradictory. On one hand, a reduction in urinary 8-iso-PGF2α was observed inversely proportional to the phenolic content of the olive oils (phenolic concentrations between 24.38 and 97.5 mg/dose) when administered in a single dose [39,40]. On the other hand, further short-term studies found no significant changes in F2-IsoPs excretion. In a crossover study with olive oil rich in phenolics conducted with 182–184 healthy volunteers, despite the high dose of phenolics tested (366 mg/kg, 25 mL daily, 21 days), the oxLDL decreased, but no such effect was observed in the IsoPs [30]. However, a significant decrease in the plasma IsoPs was observed in the above study when comparing only the baseline data and the endpoint data at the end of the crossover interventions [41]. Regarding the studies with dietary supplements, no changes in urinary levels of F2-IsoPs were recorded in young people after multiple doses of olive leaf supplements in liquid or capsule formats [42].

Although the human trials of the F2-IsoPs modulation from olive-derived foods have yielded contradictory results, it is noted that the trials above evaluated different doses, populations, timing and duration, and food matrices; in addition, different analytical methods and biological fluids were analyzed, all variables that affect the final outcome [43]. Furthermore, it is recognized that under normal conditions IsoPs appear in the plasma and urine, and their levels are only amplified by oxidative stress [44,45]. Indeed, several clinical studies that report a significant modulation of IsoPs levels after a specific treatment have been carried out in subjects showing an oxidative stress condition [36,38,46,47,48,49,50,51,52,53]; that is, the reduction of oxidative markers could be significant only if their levels are high at the beginning of the study. In contrast to this, antioxidant supplementation in subjects with a balanced redox state seems to be of little clinical relevance.

In the present study we evaluated the degree of urinary excretion of F2-IsoPs pre-intervention and after taking the food supplements only in those subjects with a high level of oxLDL at baseline. Applying this criterion, we found that both dietary supplements significantly reduced the F2-IsoPs level as soon as 0.5 h after the intake (*p* < 0.05, mean of differences 1.93 and 2.64 for IP-1 and IP-2, respectively), thus suggesting that subjects with a redox imbalance may benefit from the food supplements’ integration to protect lipids from oxidation.

Most of the studies that have evaluated the effects of the intake of olive phenols on the levels of oxLDL and/or F2-IsoPs have been carried out after interventions in the short to medium term. Our previous study [21] showed that HT administered with these watery food supplements is absorbed from the intestinal tract and is rapidly metabolized to phase I and phase II metabolites. The major metabolites of HT (i.e., homovanillic acid, hydroxytyrosol 3-O-sulfate, and 3,4-dihydroxyphenylacetic acid) peaked in the blood at 30 min after intake and were excreted in the urine as early as 30 min and up to 12 h post-intake [21]. We believe that the effects on oxLDL and F2-IsoPs may be directly linked to HT absorption, distribution, metabolism, and excretion. This concept is supported by previous data obtained on the bioavailability of HT administered with these supplements. In fact, the lipoxidation markers correlate with the metabolic products of HT over time. For example, in the blood the oxLDL correlates negatively with the levels of HT-3-glucuronide (significance level < 0.05), and urine levels of entPGF2α correlate negatively with HT-3-sulfate (significance level < 0.05). In addition, we here report for the first time lipoxidation-reducing effects in vivo as early as 30 and 60 min after intake of food supplements containing HT as the main bioactive.

## 4. Materials and Methods

### 4.1. Standards and Reagents

Citric acid, phosphoric acid, L(+)-ascorbic acid, 1-butanol, and ethyl acetate were from Roth (Karlsruhe, Germany). Oxidized LDL ELISA kits (Cod: 10-1143-01) were from Mercodia (Uppsala, Sweden). The 9α,11α,15S-trihydroxy-prosta-5Z,13E-dien-1-oic-3,3,4,4-d4acid (PGF2α-d4, CAS 34210-11-2), 9β,11β,15R-trihydroxy-(8β,12α)-prosta-5Z,13E-dien-1-oic acid (ent-PGF2α, CAS 54483-31-7), 9α,11α,15S-trihydroxy-(8β)-prosta-5Z,13E-dien-1-oic acid (8-iso PGF2α, CAS 27415-26-5), and (3Z)-5-[(1S,2R,3R,5S)-3,5-dihydroxy-2-[(1E,3S)-3-hydroxy-1-octen-1-yl]cyclopentyl]-3-pentenoic acid (2,3-dinor-8-isoPGF2α, CAS 221664-05-7) were purchased from Cayman Chemical (Ann Arbor, MI, USA). Formic acid was from Merck (Darmstadt, Germany). LC-MS-grade water and methanol were purchased from VWR Chemicals (Darmstadt, Germany).

### 4.2. Investigational Products (IPs)

The liquid food supplements were provided by Fattoria La Vialla S.A.S (Castiglion Fibocchi, Arezzo, Italy). They are derived from olive fruit (*Olea europaea* L.) vegetation water subjected to concentration, reverse osmosis, and filtration; commercial brands are *Oliphenolia bitter* (IP-1) and *Oliphenolia* (IP-2). IP-1 consists of a 94% concentrated vegetation water and 6% concentrated lemon juice (*Citrus limon* L. fructus). IP-2 is composed of 30% further concentrated vegetation water and 70% grape juice (*Vitis vinifera* L. fructus). Quantification of HT and derivatives in the IPs was conducted as previously described [20]. 

### 4.3. Study Design

The study protocol was approved by the Ethics Committee of the State Medical Association of Rheinland-Pfalz (Mainz, Germany). The clinical study was conducted as set out in the Code of Ethics of the World Medical Association (Declaration of Helsinki) by *daacro GmbH & CO* at the Science Park Trier (Germany) and is registered at ClinicalTrials.gov (identifier: NCT04876261). 

The results presented here are secondary outcomes of a study that investigated the bioavailability of HT after acute intake of IP-1 and IP-2 in healthy men, compared with EVOO [21]. The study design was single-blind, randomized, single-dose, three-way cross-over, in which volunteers ingested different concentrations of olive phenolics through a single dose of the corresponding IPs. IPs were orally administrated together with 200 mL of water after an overnight fast of at least 10 h. Water intake was allowed ad libitum, and a controlled basal diet was administered 2 h after intake.

### 4.4. Participants

Twelve healthy male volunteers were recruited who met the inclusion and exclusion criteria (age 21–50, BMI > 18.5 < 29.9 kg/m^2^, nonsmokers, no eating disorder, no drug treatment in the previous or ongoing 2 weeks, no intake of food supplements, and no drug or alcohol abuse). Written informed consent was obtained from all participants prior to starting the trial. Diet indications included to avoid consuming olive-derived products as well as alcohol and supplements with HT, vitamins, minerals, and antioxidants 2–4 days before the first intake and during the whole study. Volunteers underwent a wash-out period of 6 days between the interventions to avoid interference between the IPs. In addition, three days prior to and at each intervention, volunteers avoided moderate or intense physical activity.

### 4.5. Sampling

At each intervention visit, a baseline blood sample was collected immediately before the administration of the IP. Further six blood samples were collected 0.5, 1, 1.5, 2, 4, and 12 h after the intake. EDTA-plasma samples were obtained and stored at −80 °C until analysis.

At each intervention visit, a baseline urine sample was collected from −240 to 0 min before the administration of the IP. Further six urine samples were collected after the intervention from 0 to 30 min, 30 min to 1 h, 1 to 1.5 h, 1.5 to 2 h, 2 to 4 h, and 4 to 12 h. The total volumes excreted were measured, stabilized with 1.88 g/L of ascorbic acid, and stored at −80 °C until analysis.

### 4.6. Analysis of Plasma oxLDL

The plasma samples were thawed at room temperature and immediately quantified for oxLDL with a sandwich ELISA assay according to the manufacturer’s recommendations. Briefly, the sandwich assay uses two monoclonal antibodies against separate antigenic determinants of the oxidized apolipoprotein B molecule. During a first incubation the plasma oxLDL reacts with the capture antibody mAb-4E6. The next steps include incubation with the peroxidase-conjugated secondary anti-human apolipoprotein B antibody, which is detected spectrophotometrically by reaction with 3,3′, 5,5′-tetramethylbenzidine. The oxLDL concentration was recorded in duplicates in a Fluostar OPTIMA reader (BMG Labtech, Offenburg, Germany) and calculated with a five-parameter logistic (5PL) curve and automatic weighting using 1/Y^2^. The mean values of oxLDL, expressed in units per liter (U/L), were used for the statistical analysis.

### 4.7. Analysis of Urinary F2-IsoPs

The isomers 8-isoPGF2α, ent-PGF2α, and 2,3-dinor-8-isoPGF2α were evaluated with UHPLC-DAD-MS/MS following an internal method based on unpublished work. In brief, urine samples were thawed and spiked with aqueous formic acid and PGF2α-d4. Extraction solvent (5% butanol in ethyl acetate) was added, mixed, and placed in an ice bath for 3 min, followed by centrifugation. The organic phase was collected and evaporated under nitrogen flow. The extraction step was repeated twice. The dry sample was resuspended with methanol and formic acid, filtered through 0.2 µm regenerated cellulose filters (Macherey Nagel, Düren, Germany), and transferred to a new vial.

Measurement was conducted with an Acquity UPLC I-Class system coupled to a XEVO-TQS micro mass spectrometer (Waters, Milford, MA, USA) using standard substances as reference. The instrument consisted of a sample manager cooled to 10 °C, a binary pump, a column oven, and a diode array detector. The column oven temperature was set to 40 °C. Eluent A was acetonitrile with 0.1% formic acid, eluent B was water with 0.1% formic acid, and the flow was 0.4 mL/min on an Acquity BEH Shield RP18 column (150 mm × 2.1 mm, 1.7 µm particle size) combined with an Acquity BEH Shield RP18 precolumn (Acquity, 2.1 mm × 5 mm, 1.7 µm), both from Waters (Milford, MA, USA). The gradient started with 30% A and was raised to 80%. The peaks were identified with MS/MS. 

All samples were run in duplicate. Data were acquired and processed using MassLynx 4.1 (Waters, Milford, MA, USA) and normalized by dividing the concentration by the urinary creatinine content in the sample. Mean values of F2-isoP isomers, expressed in µg/g, were used for the statistical analysis.

### 4.8. Data Analysis

The average values of concentration for each of the samples were calculated in Microsoft Excel version 16.0. Average values were further processed using GraphPad version 5.00 (San Diego, CA, USA) software to represent in the graphs and table. For the raw statistics, classical statistical methods using median, mean, standard deviation, and confidence intervals were used. Data are expressed as mean ± standard error (SEM) unless otherwise indicated. Student’s paired t-test was performed to compare the results before and after intake of the IPs.

## 5. Conclusions

The present study shows that the antioxidant effects of food supplements rich in olive phenolic compounds, and especially in bioavailable HT, are promising for the reduction of lipoxidation in vivo.

The oxLDL values significantly decreased by 17.5 ± 1.83 U/L at 1 h after intake. Considering the intervention groups individually, an oxLDL reduction tendency was observed shortly after intake. In general, these results support the positive effect of olive phenolics in the reduction of lipoxidation. We further support the finding that antioxidant effects from foods high in antioxidants can be expected most in people with elevated oxidative stress. Indeed, by measuring the F2-IsoPs in urine we showed that in healthy subjects with high oxLDL levels at the beginning of the study, both food supplements significantly lowered the excretion of F2-IsoPs at 0.5 h post-intervention, remaining low until 12 h.

Overall, the results obtained indicate the efficacy of both the food supplements to reduce the level of lipoxidation shortly after intake, which is a valuable aid to prevent and combat oxidative damage in the organism. Findings from the study warrant further research for the use of these HT-rich food supplements in personalized nutrition.

## Figures and Tables

**Table 1:** Nutritional value and content of hydroxytyrosol (HT) and derivatives in one flask (25 mL) of the food supplements. Ole: oleuropein, HVA: homovanillic acid, DOPAC: 3,4-dihydroxyphenylacetic acid. n.d.: not detectable; *: according to producer.

Nutritional Value * [g/25 mL]	IP-1	IP-2
Energy (kJ)	18.75	138.75
Fat	0.00	0.01
of which saturates	0.00	0.00
Carbohydrate	1.04	8.01
of which sugars	0.26	6.88
Protein	0.05	0.09
Salt	0.01	0.005
Potassium	0.201	0.161
**HT and Derivatives (in the free forms) [mg/25mL]**		
HT	30.6	61.5
Ole	0.04	0.07
HVA	n.d	n.d
DOPAC	n.d	n.d
Total polyphenols *	274.5	252.5

**Table 2:** F2-IsoPs excretion following IP-1 and IP-2 intake. Mean content (µg/gr) ± Standard Error (SEM) is expressed as the sum of 8-isoPGF2α, ent-PGF2α, and 2,3-dinor-8-isoPGF2α. N: sample size; *: significantly different from baseline (*p* < 0.05).

Time (h)	IP-1 (n = 9)		IP-2 (n = 7)	
	Mean	SEM	Mean	SEM
0	4.593	0.88	4.797	1.11
0.5	2.680 *	0.18	1.955 *	0.26
1	3.246	0.19	2.353 *	0.27
1.5	5.259	1.22	2.815	0.39
2	4.438	0.65	2.813 *	0.39
4	3.322	0.21	2.542 *	0.30
12	2.801 *	0.24	2.757 *	0.45

## Data Availability

Not applicable.
